# Establishing diversity, equity, inclusion and justice task forces for District of Columbia area cancer centers: Lessons learned implementing efforts in oncology

**DOI:** 10.1371/journal.pone.0341662

**Published:** 2026-02-12

**Authors:** Katarina E. AuBuchon, Anne McDonnell, Hannah Arem, Teletia Taylor, Yasmine M Kanaan, Marjorie C. Gondré-Lewis, Barbara W. Harrison, Nicole P. Chapell, Julie E. Bauman, Christopher J. King, Pavani Chalasani, Christopher Gallagher, Carla D. Williams, Mandi L. Pratt-Chapman

**Affiliations:** 1 MedStar Health Research Institute, Washington, DC, United States of America; 2 The George Washington University Cancer Center, Washington, DC, United States of America; 3 Georgetown University, Washington, DC, United States of America; 4 Howard University Cancer Center, Washington, DC, United States of America; 5 Howard University College of Medicine, Washington, DC, United States of America; 6 Department of Obstetrics & Gynecology, Beth Israel Deaconess Medical Center, Boston, Massachusetts, United States of America; 7 Department of Obstetrics, Gynecology, and Reproductive Biology, Harvard Medical School, Boston, Massachusetts, United States of America; Prince Sattam bin Abdulaziz University, SAUDI ARABIA

## Abstract

**Purpose:**

To address observed cancer health inequities resulting from historically-embedded structural bias in quality, equity, and access to health care delivery, we organized and coordinated Diversity, Equity, Inclusion, and Justice (DEIJ) Task Forces across three cancer centers in the Mid-Atlantic region.

**Methods:**

We recruited multi-disciplinary, diverse faculty and staff for each task force to optimize capacity to implement sustainable, effective strategies for institutional change. We developed an educational curriculum aligned with enhanced National Culturally and Linguistically Appropriate Services (CLAS) Standards and the DC Hospital Health Equity Framework. Over 18 months, task force members were invited to 14 sessions designed to help teams assess and prioritize critical areas for improvement through organizational assessments and then create and implement action plans.

**Results:**

Task force goals focused on reducing cancer disparities by improving patient information accessibility, expanding the reach of implicit bias training for providers, and improving accurate socio-demographic data collection. The task forces made meaningful progress on two of these three goals. Challenges included competing time demands, incomplete and limited data generated by institutions, limited insight into operational protocols and inadequate resources and time to complete work.

**Conclusion:**

Clear, accessible patient-reported demographic data, protected staff time, leadership commitment, and significant institutional resource supports are critical to effectively advance DEIJ work.

## Introduction

Cancer inequities are unfair, unjust, and avoidable differences in cancer burden and outcomes that are caused by social, economic, and environmental disadvantages [1,2]. Observed cancer health inequities among historically marginalized people (e.g., Black people, LGBTQ+ people) are caused by structural classism [3], racism [4–6] sexism [7], cis-heterosexism [8,9], and their intersections. Ultimately, cancer inequities require multi-level interventions to dismantle systemic biases and address long-standing barriers to care access [10–12]. Diversity, Equity, Inclusion, Justice (DEIJ) commitments implemented in cancer centers – from the clinic to the leadership – can meaningfully advance health equity.

To address observed cancer health inequities [13–15] we organized and coordinated three DEIJ Task Forces in their respective cancer centers, each with distinct clinical environments and diverse patient populations. The task forces assessed, prioritized, and implemented strategies to address structural inequities impacting historically marginalized patients.

## Methods

This study was approved by the Georgetown University IRB (#00004397). All participants consented verbally or in writing to participate in DEIJ Task Force activities. Decisions made in the course of operations to advance DEIJ Task Force work have been summarized here.

### Framework

To build capacity for task force members, we developed a curriculum [16] aligned with enhanced National Culturally and Linguistically Appropriate Services (CLAS) Standards [17] and the District of Columbia (DC) Health Equity Framework [18]. These frameworks ensured alignment of task force goals with pre-existing hospital leadership commitments.

### Curriculum development

The curriculum was organized into eight required educational sessions and six optional technical assistance sessions (Table 1). Sessions progressed from evaluating individual-level biases to examining institutional-level policies and practices to exploring successes at other institutions initiating structural changes. Optional technical assistance sessions from external subject matter experts were available to learn from successful efforts at other institutions. All sessions were open to the public for individuals to promote collaborative information sharing [19]. All sessions were recorded and made publicly available [16].

**Table 1:** DEIJ task force educational sessions.

Session Topic	Learning Objectives
1. Introduction to DEIJ Implementation	1. Define key terminology related to diversity, equity, inclusion, and justice (DEIJ) principles, including assessment metrics. 2. Recognize opportunities for organizational improvement using the DC Health Equity Framework. 3. Assess organizational culture for racial equity through standardized measurements. 4. Describe and identify DEIJ organizational assessment metrics.
2. Culturally Appropriate Care	1. Define key terminology related to cultural humility, cultural responsiveness, and culturally appropriate care. 2. Describe culturally appropriate care for patients whose native language is not English. 3. Describe organizational culture that prioritizes the social needs of historically marginalized patient populations. 4. Assess organizational culture for cultural appropriateness through standardized measurements
3. Healthy, Equitable Communities & Community Engagement	1. Describe social determinants of health and how they affect cancer outcomes. 2. Explain key components of healthy and equitable communities. 3. Explain what reciprocal community partnerships look like. 4. Describe community engagement, continuous improvement, and accountability. 5. Assess organization’s community engagement culture through standardized measurements.
4. Communicating Equity to Stakeholders & Inclusive Governance	1. Identify strategies for disseminating institutional values to key partners. 2. Assess organizational capacity and infrastructure to communicate DEIJ values/ commitments. 3. Describe inclusive and equitable governance. 4. Assess organizational culture for inclusive and equitable governance. 5. Assess organizational inclusivity through standardized measurements.
5. Action Planning	1. Identify areas for organizational improvement based on organizational assessment (exercises from past sessions). 2. Identify action planning principles for health and equitable communities. 3. Present action plan to address priority areas for health equity advancements.
6. Action Plan Implementation	1. Present Action Plan and discuss Action Plan Implementation with peer collaboration. 2. Evaluate Action Plans to ensure they reflect CLAS Standards. 3. Evaluate Action Plans to ensure they reflect DC Health Equity pillars. 4. Evaluate Action Plans to ensure they reflect Strategic, Measurable, Ambitious, Realistic, Time-bound, Inclusive, and Equitable (SMARTIE) goals.
7. Utilizing Staff and Community Perspectives to Improve Social Needs Screening & Resource Connection*	1. Understand the impact of social needs screening on equitable care. 2. Identify and strategize improvements for social needs screenings within institutions.
8. Making the Business Case for DEIJ*	1. Describe the purpose of a business case. 2. Identify opportunities to align health equity initiatives with institution’s business priorities. 3. Generate a business case for health equity initiatives, including a basic ‘pro forma.’
9. Optimizing Race, Ethnicity, & Language (REaL) and SOGI Data Collection*	1. Understand the impact of Race Ethnicity and Language (REaL) and Sexual Orientation and Gender Identity (SOGI) patient population data. 2. Identify and strategize improvements for REaL and SOGI data collection. 3. Identify and strategize improvements for how patient data is used to understand healthcare disparities.
10. 6-Month Progress	1. Assess current progress on institutional task force priorities. 2. Collaborate to strategize steps forward when encountering institutional barriers to affecting change.
11. Developing Data-Driven Dashboards*	1. Describe importance of Sexual Orientation and Gender Identity (SOGI) data collection for all patients and communicate to leadership. 2. Identify and strategize improvements for SOGI data collection. 3. Build data dashboards to drive change and equity. 4. Understanding data validity, accuracy and accessibility issues and solutions for SOGI data.
12. Lessons Learned from Standing Up DEIJ Strategy*	1. Strategize practices and policies for health equity and workplace diversity. 2. Create and develop SMARTIE goals for internal and external workplace success.
13. Applying an Equity Framework to Patient Safety Events and Root Cause Analysis*	1. Apply equity framework to patient safety events and Root Cause Analysis (RCA). 2. Assess patient safety events with an equity framework.
14. Conclusions	1. Assess progress on institutional task force priorities. 2. Re-assess organizational community engagement culture, and identify progress given task force involvement.

DEIJ = Diversity, Equity, Inclusion, and Justice.

*indicates optional sessions.

### Task force development

Institutional Principal Investigators (PIs) identified five to eight multidisciplinary champions from diverse occupational backgrounds (e.g., research, clinical) and roles (e.g., physicians, nurse managers, community health workers, research associates) to join their respective institutional task force. By recruiting task force members from diverse backgrounds and roles, we aimed to identify individual blind spots and implement sustainable and effective strategies for institutional change.

### Development of DEIJ goals

In July 2023, each task force began an organizational assessment (see Table 2) which guided the task forces’ action plan development. Each task force developed a 12-month action plan with goals based on needs identified during the organizational assessment.

**Table 2:** Select questions from organizational assessment.

DEIJ Practices	Culturally Appropriate Care	Community Engagement	Inclusive Governance
How does your cancer center track racial equity performance, monitor progress and ensure accountability to advance DEIJ?	What is your cancer center’s process for testing literacy level of printed patient materials?	Is there motivation among leadership, clinicians, researchers, and staff of the cancer center to explore how to redeploy assets as a means of building the community’s capacity to address racial inequities? Please explain.	What type of training does your cancer center provide to address implicit bias and cultural humility for all associates? Is there a demonstrated commitment to training that progresses in complexity on these topics over time?
How are institutional goals to reduce barriers to opportunity and racial disparities reflected in resource allocations?	What measurable goals does your cancer center have regarding cultural and linguistic competence?	Does your cancer center regularly provide information to the public through print materials and activities highlighting your efforts to provide culturally responsive care to all patients/clients? How so?	How does your cancer center address staff and clinician bias (e.g., public health insurance, race, ethnicity, sexual orientation, gender identity, language stigma)?
How does your cancer center address formal grievances and complaints from patients? Is the process inclusive and affirming of all patients?	Does your cancer center systematically provide language assistance services at no cost to patients who do not prefer English as a language?	Has your cancer center developed ways to invest in the community (e.g., hospital with farmers market, childcare, renovated housing)? How so?	Does your cancer center require diversity awareness and cultural responsiveness at all levels of the organization (i.e., staff, management, providers, etc.)? Please explain.

Task forces refined goals into specific, measurable, achievable, relevant, time-bound, inclusive, and equitable (SMARTIE) goals, which evolved over time based on what was within the authority and capacity of task force members (see Table 3) [20].

**Table 3:** Evolution of task force goals and accomplishments.

	Organizational Assessment	Objectives	Barriers to Implementation	Accomplishments at 18 Months
Institution 1	Unclear how the cancer center tracks racial equity performance to advance DEIJ objectives Unclear what the commitment is to health equity priorities Unaware of the cancer center’s process for literacy level and language needs of printed materials	*Original objectives:* Map cancer continuum pathway for patients with metastatic colorectal cancer Improve communication process and care coordination between inpatient case managers and outpatient social work to reduce loss to follow up from solid tumor new diagnosis to outpatient treatment Increase care coordination with primary care clinicians to address comorbidities and chronic conditions outside of oncology Increase referrals to palliative care to reduce hospital admissions for pain and uncontrolled symptoms *Revised objective*: Create patient orientation welcome packet with resources and contact information for cervical cancer patients by June 2024	Staff turnover and attrition Competing demands (no dedicated time) No technology support: inability to assess needs because information not readily available Unclear workflows across disease sites No budget Need for community perspectives to vet materials and advance the work	Focused on gaining institutional buy-in by engaging leadership to integrate DEIJ task force goals with the institution’s goals Pivoted from original goals to focus on attainable goals in creating an introductory information packet to give to patients at initiation of cancer treatment Collaboration across multiple groups and passion in advancing the work in the face of roadblocks
Institution 2	Patient language, ethnicity, sexual orientation, gender identity, and disability are collected during intake and registration There are no measurable goals set for cultural and linguistic competence. For some activities, materials are written in different languages (flyers, brochures, etc.) There is no requirement for implicit bias training and cultural humility in the cancer center	*Original objectives:* Ensure language needs are met for cancer center patients and research materials	Unable to access sociodemographic data to identify needs, delays in getting this information Time to process requests for language translation No budget for patient document translation and signage Competing demands (no dedicated time)	Identified needs in website changes and point of contact improvements on the website; updated signage in hospital and cancer center Added seminar to integrate language services into research Met with the DEIJ office for the university affiliated with the institution to measure steps being done and still being missed Provided cultural awareness activities for all affiliated staff, students, and faculty
		Assess patient experiences and examine socio-demographic factors		
		Expand diversity training for medical students, faculty, and staff *Revised objectives:* Post signs for interpreter services for research participants at hospital entrance by April 2024 Update hospital multi-cultural affairs web site by April 2024 Request report of patient satisfaction		
Institution 3	Most patient-facing information is pulled from central repositories (e.g., American Cancer Society), but there is no confirmation of literacy level No current goals regarding cultural and linguistic competence besides annual web-based training Printed materials are not institutionally branded and culturally responsive to the patient population	*Original objectives:* Expand annual training on implicit bias and inequity Demonstrate need for dedicated patient advocacy positions Explore opportunities to invest in technology and expand use of technology to facilitate identification of patient needs and impact of social determinants of health Create formal system to review educational information that is given to patients and determine what needs to be improved and how; expand languages in which educational materials are offered *Revised objectives:* Meet with patient support team to identify top patient information concerns by talking with nurse navigators by March 2024 Create Amharic video to orient patients in collaboration with nurse navigator by June 2024. Conduct interviews with patients to inform one-pagers by October 2024. Draft patient-facing one pager with key cancer care terms by June 2024.	Task force attrition Competing demands (no dedicated time) Lack of centralized, accessible data Competing implicit bias training required by institutional leadership	Met with patient support team to identify top information needs of patients Identified need for standardized patient materials Drafted materials for navigators to use, and plan to incorporate patient perspectives on materials prior to implementation Built on past work from a navigator who had Amharic materials Brought together components of the cancer center that previously were disconnected.

### Peer learning approach

Each task force shared their action plan with and obtained feedback from peer task forces. The three task forces convened at 6, 12, and 18 months for cross-institutional sharing to discuss their progress and share lessons learned.

## Results

Action plans prioritized improving patient information accessibility, expanding the reach of implicit bias training, and improving accurate socio-demographic data collection to assess inequities. Overall, SMARTIE goals evolved over time (see Table 3). Progress was made in improving accessibility of patient information and expanding reach of implicit bias training, but little progress was made on improving accurate socio-demographic data collection.

### Improving patient information accessibility

All three task forces improved accessibility of patient-facing materials by creating new patient information resources, lowering the reading level of existing resources, and/or translating materials into additional languages. Institution 1 developed an introductory packet to provide to patients at the initiation of cancer treatment and began work on disease-specific information packets for patients. Institution 2 identified major language needs. Working with their institutional DEIJ office, they created an expedited assessment and review process for materials, revised the institution’s website to direct people to language services, and hosted an educational seminar on language services available within the institution. This work resulted in improved integration of interpretation services across departments. Institution 3 worked with nurse navigators to develop a standardized information sheet with definitions of common cancer-related terminology for patients.

### Implicit bias training

All three institutions expanded implicit bias training for cancer center staff and providers [21]. Institutions 1 and 2 attained a high training completion rate, but institution 3 was challenged by receiving notice of a competing implicit bias training that was made mandatory at their institution. Because the rollout of the mandatory training was independent from the task force, the total percentage of clinicians and staff that completed any kind of implicit bias training at institution 3 was unavailable. Thus, while the completion rate was low for the task force provided training, exposure to some bias training was likely higher.

### Improving accurate socio-demographic data

Data gaps led to incomplete organizational assessments at the very beginning of the 18-month quality improvement process. These challenges led the task forces to try to improve accessibility of accurate socio-demographic data. However, little progress was made on this goal due to lack of task force authority to change data collection processes, workflows, and relevant medical record forms.

## Discussion

There is an urgent need to advance cancer health equity in the US [22]. Our quality improvement project contributes to the literature by documenting accomplishments and challenges in implementing DEIJ initiatives in clinical cancer care environments. Though many academic medicine DEIJ initiatives focus on a sense of belonging for historically marginalized faculty and staff [23], and increasing knowledge of systemic injustices [24–26], our task forces specifically sought to improve equity for patients in the context of cancer care delivery.

A major overall accomplishment of the task forces was to infuse DEIJ efforts into operations and higher-level discussions at three University-affiliated cancer centers in Washington, DC. DEIJ initiatives at other US academic institutions are often led by assistant professors [26]; however, we were able to recruit leaders across multiple levels of influence (administrative, patient care, research) to collaborate on our DEIJ task forces. Across the three institutions, steps were taken toward advancing CLAS standards in practice and integrating DEIJ values into institutional culture. Our focus on patient education and quality improvement aligns with needs identified in the literature. For example, in a study of individuals initiating DEIJ work in radiation oncology, 23 of 34 (76%) respondents expressed that patient education was a primary focus [26].

Lessons learned included the importance of leadership engagement and buy-in and the critical need for protected time and designated staff to advance specific DEIJ work. DEIJ champions in other institutional settings have reported challenges such as fatigue from individuals spearheading initiatives in their institutions, limited time/competing demands and lack of leadership buy-in [27]. Our experience suggests that providing protected time for staff whose roles align with and are rewarded by DEIJ work can contribute to more rapid achievement of DEIJ goals. Additionally, we found that when DEIJ principles were centered in an organization’s mission, requests for increased resources and staff to advance DEIJ goals were more successful. Integrating goals with quality improvement requirements for cancer committee accreditation also facilitated greater success.

Barriers to success included limited human resources (e.g., staff turnover, competing demands) and institutional support (e.g., data access, protected time). For institutions 1 and 3, DEIJ work was not explicitly rewarded. Without clear resources, protected time or institutional incentives, members at institutions 1 and 3 were unable to devote the effort they wished to achieve their team goals, reporting structural barriers to success related to capacity and commitment. Members at these two institutions reported an underlying perception that DEIJ work is approved, but pragmatically un-supported (e.g., protected time). Staff turnover slowed down task force work at these two institutions. Our experience aligns with previously reported limitations to DEIJ work documented in the literature. For example, among 50 radiation oncology departments with DEIJ initiatives, only 38.2% of DEIJ leaders had administrative support, only 29.4% had funding, and only 23.5% had protected time [26].

All task forces faced barriers with hierarchal, bureaucratic departments and structural or cultural barriers, as well. For example, institution 1 reported a heavily siloed structure that impeded cross-departmental collaboration. Institution 2 struggled to identify patient information needs, as patient materials were not clearly identifiable and organized for sharing with the task force. Additionally, institution 2 reported issues assessing patient experience disparities due to limited access to patient socio-demographic data, creating a “no data, no problem” problem.

Despite these obstacles, each institution made significant progress by refining their objectives to focus on more attainable goals and fostering collaboration across departments. The success of the task forces was driven by the dedication, enthusiasm, motivation, and cross-departmental collaboration of its members. This propelled progress and energized efforts. All task forces were committed to sustaining DEIJ work through collaborative efforts, expanded engagement of clinical leadership, and improved institutional alignment.

This study is limited to the experiences of only three cancer centers clustered in one geographic area. However, sharing these lessons will be helpful for institutions seeking structural change in other institutional and geographic settings. Other clinical settings wishing to improve DEIJ can leverage our lessons learned by obtaining early leadership buy-in, integrating manageable DEIJ goals within institutional strategic imperatives, distributing tasks among task force members with the authority and capacity to advance group goals, and rewarding DEIJ work in practice through workload reductions, dedicated time, and other recognition aligned with institutional values.

While we discussed at great length whether to include patient representatives in each task force, we opted to forgo patient representatives to encourage candor around internal challenges. Yet, this does limit our perspective to that of internal team members at each cancer center. Future work should, when possible, include patient perspectives to ensure meaningful improvements align with patient needs. Future work could also consider establishing a platform for community collaboration where task force members can share resources, learnings, and best practices with community members [22]. Collaboration with external community organizations may provide valuable support and motivation to overcome challenges.

Finally, while the findings of our assessments and action plans are specific to the institutions involved in our study, the educational recordings of task force sessions and affiliated resources of sessions are published and accessible to all on the www.cancercontroltap.org technical assistance web site [16]. Resources include downloadable templates that other institutions may use to conduct their own organizational assessment and action planning.

## Conclusion

Developing a multi-disciplinary task force to advance DEIJ goals created meaningful improvements in our study. However, strong institutional leadership buy-in, alignment with strategic priorities, protected time and resources, and authority to advance structural change are critical to build an inclusive organizational culture and operationalize DEIJ values into policies and practice.

## References

[1] National Cancer Institute. Cancer Disparities. https://www.cancer.gov/about-cancer/understanding/disparities. 2024.

[2] AACR Cancer Disparities Report 2024. American Association for Cancer Research. 2024. https://cancerprogressreport.aacr.org/disparities/

[3] Levit LA, Byatt L, Lyss AP, Paskett ED, Levit K, Kirkwood K, et al. Closing the rural cancer care gap: three institutional approaches. JCO Oncol Pract. 2020;16(7):422–30. doi: 10.1200/OP.20.00174 32574128

[4] Best AL, Roberson ML, Plascak JJ, Peterson CE, Rogers CR, Hastert TA, et al. Structural racism and cancer: calls to action for cancer researchers to address racial/ethnic cancer inequity in the United States. Cancer Epidemiol Biomarkers Prev. 2022;31(6):1243–6. doi: 10.1158/1055-9965.EPI-21-1179 35642391 PMC9306268

[5] King CJ, Buckley BO, Maheshwari R, Griffith DM. Race, place, and structural racism: a review of health and history in Washington, D.C. Health Aff (Millwood). 2022;41(2):273–80. doi: 10.1377/hlthaff.2021.01805 35130070

[6] Gondré-Lewis MC, Abijo T, Gondré-Lewis TA. The opioid epidemic: a crisis disproportionately impacting black americans and urban communities. J Racial Ethn Health Disparities. 2023;10(4):2039–53. doi: 10.1007/s40615-022-01384-6 36068482 PMC9447354

[7] Keenan BP, Barr E, Gleeson E, Greenberg CC, Temkin SM. Structural Sexism and Cancer Care: The Effects on the Patient and Oncologist. Am Soc Clin Oncol Educ Book. 2023;43:e391516. doi: 10.1200/EDBK_391516 37155944

[8] Matthews AK, Breen E, Kittiteerasack P. Social determinants of LGBT cancer health inequities. Semin Oncol Nurs. 2018;34(1):12–20. doi: 10.1016/j.soncn.2017.11.001 29373163

[9] Flores AR, Conron CJ. Adult LGBT Population in the United States. The Williams Institute. 2023. https://williamsinstitute.law.ucla.edu/wp-content/uploads/LGBT-Adult-US-Pop-Dec-2023.pdf

[10] Arnold LD, McGilvray MM, Kyle Cooper J, James AS. Inadequate Cancer Screening: Lack of Provider Continuity is a Greater Obstacle than Medical Mistrust. J Health Care Poor Underserved. 2017;28(1):362–77. doi: 10.1353/hpu.2017.0028 28239007 PMC5483852

[11] Nonzee NJ, Ragas DM, Ha Luu T, Phisuthikul AM, Tom L, Dong X, et al. Delays in cancer care among low-income minorities despite access. J Womens Health (Larchmt). 2015;24(6):506–14. doi: 10.1089/jwh.2014.4998 26070037 PMC4490771

[12] Semprini JT, Biddell CB, Eberth JM, Charlton ME, Nash SH, Yeager KA, et al. Measuring and addressing health equity: an assessment of cancer center designation requirements. Cancer Causes Control. 2023;34(Suppl 1):23–33. doi: 10.1007/s10552-023-01680-4 36939948 PMC10512189

[13] Office of Health D. District of Columbia Cancer Control Plan 2022-2026. 2022. https://dchealth.dc.gov/sites/default/files/dc/sites/doh/service_content/attachments/FINAL%20Reduced%20District%20of%20Columbia%20Cancer%20Control%20Plan_2022-2026_r6_verB_0.pdf

[14] Chandler JP, Phillips J. Racial, Education & Income Segregation in the District of Columbia. District of Columbia Office of Planning. 2020. https://planning.dc.gov/sites/default/files/dc/sites/op/page_content/attachments/Segregation%20Report%2011-18-20%20FINAL.pdf

[15] Hunger Solutions DC. Still Minding the Grocery Gap: 10th Anniversary Grocery Store Report. 2020. https://www.dchunger.org/wp-content/uploads/2021/01/StillMindingGroceryGap.pdf

[16] GW Cancer Center. Diversity, Equity, Inclusion, and Justice Implementation Toolkit. 2024. https://cancercontroltap.org/news/diversity-equity-inclusion-and-justice-implementation-toolkit

[17] U.S. Department of Health and Human Services. National Standards for Culturally and Linguistically Appropriate Services (CLAS) in Health and Health Care. https://thinkculturalhealth.hhs.gov/clas. 2023.

[18] M.L. “Session 1: Orientation and kickoff for diversity, equity, inclusion, justice, task forces.” GW Cancer Center TAP Diversity, Equity, Inclusion and Justice Implementation Toolkit. Available at https://cancercontroltap.org/news/diversity-equity-inclusion-and-justice-implementation-toolkit/

[19] Fingrut W, Beck LA, Lo D. Oncology communities of practice: insights from a qualitative analysis. Curr Oncol. 2018;25(6):378–83. doi: 10.3747/co.25.4088 30607112 PMC6291282

[20] SMARTIE Goals Worksheet. Management Center T. https://www.managementcenter.org/resources/smartie-goals-worksheet/ 2021.

[21] RosenbergD, TaylorT, AremH, DavisK, WilliamsC, BaumanJE, et al. Implementation of a Healthcare Implicit Bias Training across Three Oncology Care Settings. J Cancer Educ. 2025;:10.1007/s13187-025-02779–9. doi: 10.1007/s13187-025-02779-9 41275457

[22] Alcaraz KI, Wiedt TL, Daniels EC, Yabroff KR, Guerra CE, Wender RC. Understanding and addressing social determinants to advance cancer health equity in the United States: A blueprint for practice, research, and policy. CA Cancer J Clin. 2020;70(1):31–46. doi: 10.3322/caac.21586 31661164

[23] Yoder SR, Lonstein AB, Sharma A, Garcia-Munoz J, Moreno R, Chen AY, et al. PEARLS (Perspectives on Equity Advancement: Research and Learning Symposium), a Case Report in Promoting DEI in a Medical School Setting. Education Sciences. 2022;12(9):586. doi: 10.3390/educsci12090586

[24] Bersted KA, Lockhart KM, Yarboi J, Wilkerson MK, Voigt BL, Leonard SR, et al. A path toward equity and inclusion: establishing a dei committee in a department of pediatrics. J Clin Psychol Med Settings. 2023;30(2):342–55. doi: 10.1007/s10880-022-09929-x 36462109 PMC9735055

[25] Nabhan ZM, Scott N, Kara A, Mullis L, Dams T, Giblin M, et al. Advancing equity in graduate medical education recruitment through a diversity equity and inclusion (DEI) toolkit for program directors. J Med Educ Curric Dev. 2023;10:23821205231203136. doi: 10.1177/23821205231203136 37822778 PMC10563453

[26] Paradis KC, Franco I, Beltrán Ponce S, Chaurasia A, Laucis AM, Venkat P, et al. The current state of departmental diversity, equity, and inclusion efforts within us academic radiation oncology departments. Int J Radiat Oncol Biol Phys. 2023;116(2):219–28. doi: 10.1016/j.ijrobp.2022.06.071 36306980

[27] Esparza CJ, Simon M, London MR, Bath E, Ko M. Experiences of leaders in diversity, equity, and inclusion in US academic health centers. JAMA Netw Open. 2024;7(6):e2415401. doi: 10.1001/jamanetworkopen.2024.15401 38869901 PMC11177162

